# Clinical Performance of the BlockBuster Laryngeal Mask Airway for Blind Endotracheal Intubation in Adult Elective Surgeries: A Prospective Observational Study

**DOI:** 10.7759/cureus.98853

**Published:** 2025-12-09

**Authors:** Hariharan Lyla Manojji, Annu Theagrajan, Vidhya Narayanan

**Affiliations:** 1 Anesthesiology and Critical Care, Sree Balaji Medical College, Bharath Institute of Higher Education and Research, Chennai, IND

**Keywords:** airway, anaesthesia, emergency, intubation, laryngeal mask, supraglottic airway device

## Abstract

Introduction: Effective airway management is central to safe anesthesia practice. While endotracheal intubation remains the gold standard, supraglottic airway devices (SGADs) provide less invasive alternatives. The BlockBuster® Laryngeal Mask Airway (BBLMA) has been designed to facilitate blind endotracheal intubation while maintaining a secure airway. However, data on its performance in Indian patients are limited.

Materials and Methods: This prospective study was conducted on 60 ASA I-II adult patients undergoing elective surgery under general anesthesia at Sree Balaji Medical College and Hospital, Chennai (August 2024-August 2025). Following standard induction, the BBLMA was used as a conduit for blind intubation. The success rate, time required for intubation, ease of intubation, and hemodynamic parameters were recorded. Data were analyzed using R software. Repeated-measures ANOVA and appropriate univariate tests were applied, with a significance level of p< 0.05.

Results: Successful intubation through BBLMA was achieved in 58 of 60 patients (96.7%). Most intubations (76.7%) were graded as easy, with a mean intubation time of five minutes and 15 seconds. Hemodynamic parameters (systolic BP, diastolic BP, heart rate) showed statistically significant increases following insertion and intubation, although all remained within clinically acceptable limits.

Conclusion: The BBLMA demonstrated high success, ease of use, and hemodynamic stability during blind endotracheal intubation. These findings suggest that BBLMA can be safely considered as a first-line airway device for elective and potentially emergency anesthesia settings.

## Introduction

Securing and maintaining a patent airway is a cornerstone of anaesthetic practice, vital for ensuring adequate oxygenation and ventilation during surgical procedures. Among the various airway management techniques, endotracheal intubation remains the definitive method for establishing a protected airway [1,2]. It effectively prevents hypoxia and aspiration but may lead to airway trauma, hemodynamic stress, and other complications, particularly in patients with difficult airway anatomy [3,4].

The introduction of supraglottic airway devices (SGADs) in 1981 revolutionized airway management by offering less invasive and more rapidly deployable alternatives [5,6]. These devices were initially developed as rescue tools for unexpected difficult airways but have since evolved to include advanced models capable of providing both ventilation and a conduit for tracheal intubation [7,8]. The BlockBuster® Laryngeal Mask Airway (BBLMA), introduced in 2012, represents one such second-generation SGAD that combines the benefits of conventional laryngeal mask airways with the added advantage of facilitating blind endotracheal intubation [9,10]. It features an anatomically contoured airway tube, an integrated gastric access channel, and high oropharyngeal sealing pressures, contributing to improved ventilation and reduced aspiration risk.

Although several studies have demonstrated the global clinical utility of the BBLMA, evidence regarding its efficacy and safety among Indian patients remains limited, where anatomical variations and practice conditions can influence device performance [11,12]. Research comparing the BBLMA with other intubating laryngeal mask airways has primarily involved Western populations, restricting the generalizability of those findings [13,14]. The development of second-generation supraglottic airway devices has significantly improved airway management safety and versatility, particularly in low-resource settings [15].

Therefore, the present study aimed to evaluate the success rate, ease of intubation, time required for intubation, and hemodynamic responses associated with the BBLMA in adult patients undergoing elective surgery under general anaesthesia. Based on prior evidence and the device’s design characteristics, we hypothesized that the BBLMA would achieve a high intubation success rate with minimal maneuvering requirements and would produce hemodynamic changes that remain within clinically acceptable limits.

## Materials and methods

Study setting and duration

A prospective, observational study was conducted between August 2024 and August 2025 at Sree Balaji Medical College and Hospital, Chennai.

Ethical considerations

The study was approved by the Institutional Ethics Committee of Sree Balaji Medical College and Hospital and prospectively registered with the Clinical Trial Registry of India (CTRI/2024/07/071153). Written informed consent was obtained from all participants, and confidentiality was maintained.

Inclusion and exclusion criteria

Sixty ASA I or II adults aged 18-65 years, scheduled for elective surgery under general anaesthesia, were included. Key exclusion criteria were: ASA III/IV, anticipated difficult airway, mouth opening <2 fingers, body weight <30 kg or >90 kg, and lack of consent.

Procedure

All patients received standard anesthetic premedication with glycopyrrolate (0.01mg/kg), midazolam (0.01-0.05 mg/kg), and ondansetron (0.1 mg/kg) to ensure patient comfort and minimize intraoperative complications. Monitoring was initiated in the preoperative area and continued throughout the procedure, comprising continuous electrocardiography, non-invasive blood pressure monitoring, pulse oximetry, capnography, and temperature monitoring. Preoxygenation was done using 100% oxygen for 3 minutes. Induction of general anesthesia was carried out with intravenous fentanyl (2-3 mcg/kg) and propofol (1.5-2.5 mg/kg). Following confirmation of loss of consciousness, a non-depolarizing neuromuscular blocking agent such as vecuronium (0.1mg/kg loading dose and 0.01 mg/kg maintenance dose) or atracurium (0.5 mg/kg loading dose and 0.1mg /kg maintenance dose) was administered to facilitate muscle relaxation. The choice between vecuronium and atracurium was based on institutional practice, patient-specific factors, drug availability, and anesthesiologist preference. Both agents produce comparable neuromuscular relaxation adequate for BBLMA placement, and their use did not influence study outcomes. After achieving adequate neuromuscular blockade, the appropriately sized BBLMA (size 3 for 30-50 kg, size 4 for 50-70 kg, and size 5 for 70-90 kg) was inserted by experienced anesthesiologists. The posterior surface of the device was lubricated with a water-soluble gel to facilitate atraumatic insertion. Proper placement of the BBLMA was confirmed by observing bilateral chest rise, auscultation of breath sounds, and the presence of square wave capnography indicating correct alignment with the laryngeal inlet. Blind endotracheal intubation was then attempted through the conduit of the BBLMA using an endotracheal tube specifically designed for compatibility with the device.

The procedure was carried out in a stepwise manner. In the initial attempt, intubation was performed without any maneuvers. If this was unsuccessful, the Chandy Maneuver I was employed, involving gentle advancement and manipulation of the LMA to optimize alignment with the glottis. If intubation was still unsuccessful, Chandy Maneuver II was utilized, which involved further repositioning and rotation of the device. If all three attempts failed, the BBLMA was removed, and the patient was ventilated with 100% oxygen via mask until oxygen saturation exceeded 98%. Intubation was then completed using conventional direct laryngoscopy. The timer was initiated at the moment the BBLMA was taken in hand. Following LMA insertion, its placement was confirmed by the appearance of three consecutive capnography waveforms. Subsequently, an endotracheal tube (ETT) of appropriate size was introduced through the BBLMA. The procedure end time was recorded after confirming bilateral air entry and the presence of three consecutive capnography waveforms, indicating successful tracheal intubation. The intubation timer started when the BBLMA was taken in hand and stopped after confirmation of successful tracheal intubation through three consecutive capnography waveforms. LMA insertion time was not measured separately and was included within the total intubation time

Hemodynamic parameters, including systolic and diastolic blood pressure, heart rate, and oxygen saturation, were recorded at three critical junctures: before LMA insertion, after LMA insertion, and after successful intubation.

Sampling technique

Convenience sampling was adopted to enroll participants, selecting patients who met the defined inclusion criteria and were readily available during the study period. We acknowledge that convenience sampling may introduce selection bias; however, it was chosen due to feasibility constraints in the operating theatre environment and the observational nature of the study.

Outcome measures

The study's primary outcome was the success rate of blind intubation using the BBLMA, defined as confirmed endotracheal tube placement verified through capnography and bilateral chest expansion. Secondary outcomes included the total time taken for successful intubation, the number of intubation attempts, and the ease of intubation. The ease was graded subjectively based on the number and nature of maneuvers required, ranging from very easy (no maneuvers) to difficult (requiring conventional laryngoscopy). Hemodynamic parameters, including systolic and diastolic blood pressure and heart rate, were recorded at three critical junctures: before LMA insertion, after LMA insertion, and after successful intubation. 

Ease of intubation was graded based on the maneuvers required during the procedure: it was considered very easy when no additional maneuvers were needed, easy when only minimal maneuvers were required, moderate when multiple maneuvers were necessary, and difficult when blind intubation was unsuccessful and conventional laryngoscopy had to be used

Data collection and recording

All data were captured using a standardized proforma and entered into an electronic database (Appendix A). Variables documented included demographic information, airway assessment findings, procedural details, intubation outcomes, time metrics, and hemodynamic changes. Continuous variables such as age and time for intubation were expressed as mean and standard deviation or median and interquartile range, depending on data distribution. Categorical variables such as intubation success were summarized using frequency and percentage.

Statistical analysis

Data were analyzed using R statistical software (R Core Team, version 4.2.3). Associations were tested using the Chi-square/Fisher’s exact test for categorical variables and the t-test/Mann-Whitney U test for continuous variables. Continuous variables were summarized as mean±SD or median (IQR), and categorical data as frequencies and percentages. Data normality was assessed using the Shapiro-Wilk test, following which appropriate parametric or non-parametric tests were selected. Associations were tested using the Chi-square/Fisher’s exact test for categorical variables and the t-test/Mann-Whitney U test for continuous variables. Repeated-measures ANOVA evaluated hemodynamic changes across time points. A p< 0.05 was considered statistically significant.

## Results

Overall, 60 participants were recruited for the study. Table 1 presents the demographic characteristics of the study participants. The majority (33 participants, 55.0%) were aged ≥37 years, while 27 (45.0%) were aged <37 years. The age groups were categorised using the median age (37 years) of the study population to ensure an even distribution and meaningful comparison between younger and older participants. Female participants comprised 58.3% of the study group, while males constituted 41.7%.

**Table 1:** Socio-demographic profile of the participants (N=60).

Age distribution		
Age (in completed years)	Frequency (n)	Percentage (%)
<37	27	45
≥37	33	55
Gender distribution		
Gender		
Male	25	41.7
Female	35	58.3

Table 2 demonstrates the distribution of participants based on the success rate through BBLMA. The BBLMA demonstrated a favourable intubation success rate. Successful intubation was achieved in 58 of 60 cases (96.7%), while two cases (3.3%) required conversion to conventional laryngoscopy.

**Table 2:** Distribution of participants according to the success rate through BBLMA (N=60). BBLMA: Blockbuster laryngeal mask airway.

Status of intubation	Frequency (n)	Percentage (%)
Success	58	96.7
Failure	2	3.3
Total	60	100

Table 3 shows the distribution of participants according to the ease of intubation through the BBLMA. Most intubations (46 patients, 76.7%) were rated as easy, which included cases requiring no maneuver or only minimal optimization. Ten cases (16.7%) required moderate maneuvers, while four cases (6.6%) were classified as difficult and required conversion to direct laryngoscopy.

**Table 3:** Distribution of participants according to the ease of intubation through BBLMA (N=60). Note: The easy category included intubations requiring no manoeuvre or only minimal optimisation, consistent with the grading definitions described in the methods. BBLMA: Blockbuster laryngeal mask airway.

Ease of intubation	Frequency (n)	Percentage (%)
Easy	46	76.7
Moderate	10	16.7
Difficult	4	6.6
Total	60	100

Table 4 compares the Mean±SD values of hemodynamic parameters at three time points. Following insertion of the BBLMA and subsequent intubation, systolic blood pressure and heart rate demonstrated statistically significant increases from baseline (p=0.001 for each). Diastolic blood pressure also showed a modest but significant rise (p=0.008). Repeated-measures ANOVA demonstrated significant differences across time points for systolic BP (F(2, 58)=9.72, p=0.001, η²=0.25), diastolic BP (F(2, 58)=5.12, p=0.008, η²=0.15), and heart rate (F(2, 58)=10.43, p=0.001, η²=0.26). Although these hemodynamic changes were statistically significant, they remained within clinically acceptable limits. 

**Table 4:** Mean distribution of haemodynamic parameters at different points of time (N=60). *p-value <0.05: Statistically significant, Statistical test: repeated-measures ANOVA.

Time points	SBP (mm Hg)	DBP (mm Hg)	HR (bpm)
	Mean±SD		
Before LMA insertion	120.17±10.65	76.83±8.12	79.07±9.93
After LMA insertion	127.33±13.88	79.00±9.51	85.42±12.03
After intubation	130.00±16.26	80.67±10.71	87.97±13.55
p-value*	0.001	0.008	0.001

Table 5 presents the time required for intubation using the BBLMA. The Mean±SD intubation time was 5.3±1.9 minutes, with a median (IQR) of 4.3 (3.8-6.0) minutes. The minimum and maximum recorded times were 3.4 and 12.1 minutes, respectively. The intubation time represented the total duration from the moment the BBLMA was taken in hand until successful confirmation of tracheal intubation with three consecutive capnographic waveforms. 

**Table 5:** Time taken for intubation through BBLMA (N=60). BBLMA: BlockBuster laryngeal mask airway.

Parameters	Time (minutes)
Minimum time	3.4
Maximum time	12.1
Mean ± SD	5.3±1.9
Median (IQR)	4.3 (3.8-6.0)

In Table 6, there was no statistically significant association between age or gender and the intubation success rate using the BlockBuster® LMA (p> 0.05 for both).

**Table 6:** Association of demographic factors with intubation success using the BlockBuster® LMA (N=60). *Fisher’s Exact Test.

Variable	Category	Success n (%)	Failure n (%)	p-value*
Age group	<37 years	26 (96.3)	1 (3.7)	0.885
	≥37 years	32 (97.0)	1 (3.0)	
Gender	Male	24 (96.0)	1 (4.0)	0.808
	Female	34 (97.1)	1 (2.9)	

## Discussion

Effective airway management is the cornerstone of safe anesthesia practice. SGADs have advanced remarkably over recent decades, providing less invasive and more user-friendly alternatives to conventional endotracheal intubation. Second-generation SGADs equipped with intubation capabilities, such as the BBLMA, combine ventilation and intubation functions, improving safety and versatility.

The present study evaluated the success rate, ease of intubation, time efficiency, and hemodynamic changes associated with blind endotracheal intubation via the BBLMA in adult elective surgical patients. A success rate of 96.7% was achieved, which aligns closely with Myatra et al. (2022) [16] and Kaur et al. (2024) [5], who also reported high success and minimal airway trauma when using the BBLMA. Nazir and Saxena (2024) [12] similarly observed superior success compared with the I-gel® LMA, confirming the efficacy of BBLMA in blind intubation.

Most participants in the present study demonstrated easy or moderately easy intubation, with a mean time of 5.3±1.9 minutes. These findings are consistent with Aryan Guleria et al. (2023) [17] and Girish et al. (2024) [13], who documented comparable performance metrics. The streamlined curvature and integrated airway channel of the BBLMA likely facilitate optimal glottic alignment and reduced resistance, enhancing procedural success.

A transient but statistically significant rise in systolic blood pressure and heart rate was observed after insertion and intubation. Similar hemodynamic trends were reported by V Y et al. (2023) [11] and Aryan Guleria et al. (2023) [17]. Importantly, although these changes were statistically significant, they remained within clinically acceptable ranges, indicating that the cardiovascular response to BBLMA use is expected but not clinically concerning. This attenuated stress response, compared with direct laryngoscopy, highlights its potential utility in patients with cardiovascular comorbidities. 

Comparable results were observed by Cherian et al. [18], who reported similar intubation success and airway stability with second-generation supraglottic devices such as the i-gel and Baska mask, further supporting the effectiveness of modern LMAs. In a prospective comparative study, Saxena [19] demonstrated that the BBLMA achieved a higher success rate and shorter intubation time than the ProSeal™ LMA, corroborating our findings. As noted by Wong et al. [20], fibreoptic intubation remains the gold standard for anticipated difficult airways; however, supraglottic devices like the BBLMA provide a practical, less invasive alternative in routine anesthesia practice.

The outcomes of this study are consistent with recent reports by Nazir et al. (2024) [12], Kaur et al. (2025) [14], and Sajem et al. (2024) [21], reinforcing that the BBLMA combines high success rates with safety and efficiency across varied surgical populations. When considered with contemporary evidence, these findings support the BBLMA as a safe, effective, and time-efficient conduit for blind tracheal intubation. It can serve as both a first-line and backup device in elective and potentially emergency anesthesia settings, contributing to improved airway management strategies and patient safety worldwide. Recent reviews, including that of Zhang [22], have emphasized the expanding role of supraglottic airway devices in safe and efficient airway management, aligning with the clinical implications of our study.

This study was restricted to ASA I-II adults undergoing elective surgery, excluding high-risk, geriatric, and emergency cases.The use of two different neuromuscular blocking agents (vecuronium or atracurium), although based on standard practice, may introduce minor variability, although both agents provide comparable relaxation for BBLMA placement. The small sample size (n = 60) limits external generalizability, and the subjective ease-of-intubation scale may introduce operator bias. Similar limitations were identified by Sajem et al. (2024) and Zhang et al. (2024) [21,22]. Additionally, the use of convenience sampling may introduce selection bias and limit the representativeness of the study population. Future studies should incorporate larger, more diverse populations and objective measures such as video documentation or airway pressure monitoring.

## Conclusions

The findings of this observational analytical study strongly support the clinical efficacy and safety of the BBLMA as a dependable conduit for blind endotracheal intubation in adult patients undergoing elective surgery under general anesthesia. The device achieved a high success rate with most intubations graded as easy and associated with minimal complications. The hemodynamic changes observed were statistically significant but remained within clinically acceptable limits. When interpreted alongside contemporary Indian and international studies, these outcomes reaffirm the BBLMA’s value as both a primary and backup airway management device, particularly in settings where fiberoptic or advanced visualization tools may not be readily available. However, the findings are limited to ASA I-II elective surgical patients, and caution is required before extrapolating results to high-risk, geriatric, or emergency cases. Continued device development, structured practitioner training, and inclusion of diverse patient groups in future research will be essential to strengthen evidence for broader clinical adoption.

## Appendices

Appendix A

**Table 7:** Proforma used for data collection.

1. Patient details			
Name			
Age/sex			
Date			
IP number			
Ward			
Diagnosis			
Date of admission (DOA)			
Date of surgery (DOS)			
2. Comorbidities	Duration	Current medication	
Hypertension			
Diabetes			
Coronary artery disease (CAD)			
Cerebrovascular accident (CVA)			
Bronchial asthma			
Thyroid disorder			
Seizure disorder			
Pulmonary tuberculosis			
Smoking			
Alcohol use			
Others			
3. Pre-anesthetic evaluation			
ASA classification			
PAC date			
Height			
Weight			
BMI			
Temperature			
Pulse rate			
Blood pressure			
SpO₂			
Mallampati class (MPC)			
Mouth opening			
4. Procedure details			
Starting time			
Ending time			
Time taken for intubation			
Number of attempts			
Manoeuvre used			
5. Chandy manoeuvre	Yes/No		
Chandy manoeuvre-1			
Chandy Manoeuvre-2			
Conventional laryngoscopy			
6. Ease of intubation	Mark (✔)		
Easy			
Moderate			
Difficult			
7. Hemodynamic parameters	Before LMA insertion	After LMA insertion	After intubation
BP (mmHg)			
HR (beats/min)			
SpO₂ (%)			
EtCO₂ (mmHg)			
Post-operative complications	30 min Post-extubation	12 h Post-extubation	24 h Post-extubation
Sore throat			
Hoarseness of voice			
8. Bleeding/trauma immediately after extubation	Yes	No	
Bleeding/trauma			
